# DGI recommendations for COVID-19 pharmacotherapy

**DOI:** 10.1007/s15010-020-01519-z

**Published:** 2020-10-19

**Authors:** Jakob J. Malin, Christoph D. Spinner, German Society of Infectious Diseases (DGI)

**Affiliations:** 1 Division of Infectious Diseases, Department I of Internal Medicine, University of Cologne, Cologne, Germany; 2 Faculty of Medicine, Center for Molecular Medicine Cologne (CMMC), University of Cologne, Cologne, Germany; 3 Department of Internal Medicine II, School of Medicine, University Hospital Rechts der Isar, Technical University of Munich, Munich, Germany

**Table:** 

German Society for Infectious Diseases (DGI) recommendations for COVID-19 pharmacotherapy Last updated: 19.08.2020		
*Mild-moderate disease* Symptoms of a lower respiratory tract infection No oxygen support required	No specific therapy recommended^a^	
*Severe disease* Oxygen support required (oxygen saturation ≤ 94% on room air)	Dexamethasone^b,c,d^	6 mg/day per OS or intravenous for up to 10 days
	plus Remdesivir^e,f,g^	Day 1: 200 mg intravenous loading dose Day 2–5: 100 mg intravenous per day maintenance dose Duration: 5 days, consider extension to maximal 10 days^h^
	There are no clinical data on combination therapy with remdesivir and dexamethasone available	
*Critical disease* Hypoxic respiratory failure Invasive/non-invasive ventilation High-flow oxygen therapy	Dexamethasone^b,i^	6 mg per day intravenous for up to 10 days
	plus Remdesivir^e,j^	Day 1: 200 mg intravenous (loading dose) Day 2–5 (10): 100 mg intravenous per day (maintenance dose) Duration: 5–10 days^h^
	There are no clinical data on combination therapy with remdesivir and dexamethasone available	

*CI* confidence interval

^a^Current evidence supporting the use of remdesivir in patients with mild or moderate COVID-19 (no oxygen therapy required) [1] is insufficient

^b^Off-label-use based upon preliminary results from the RECOVERY trial [2]

^c^Reduced 28-day-mortality by one-fifth in patients that require oxygen therapy (in this study oxygen saturation 92–94% on room air) without invasive ventilation [23.3% vs. 26.2%; rate ratio (RR) 0.82 (95% CI 0.72-0.94)] [2]

^d^The clinical benefit of dexamethasone demonstrated in the RECOVERY trial was plainest in patients being treated after 7 days from symptom onset [2]

^e^Conditional European authorization for patients with COVID-19 pneumonia (≥ 12 years, weighing ≥ 40 kg) that require oxygen support and have an estimated glomerular filtration rate (eGFR) > 30 ml/min [3]; When applied, close monitoring of biochemical markers for organ toxicity (in particular hepatotoxicity) is mandatory. Adverse events must be reported immediately to the Federal Institute for Drugs and Medical Devices (humanweb.pei.de) and the manufacturer Gilead Sciences. Remdesivir should be administered as intravenous infusion over 30–60 min [4]

^f^The clinical benefit of remdesivir has been demonstrated in the Adaptive COVID-19 Treatment Trial (ACTT-1 trial) irrespective of the duration of illness. The time from symptom onset to study enrollment was 6–12 days (median 9) [1]. Subgroup analyses of a previous trial showed a numerical reduction in time to recovery of median 5 days only in patients that were treated within 10 days after symptom onset [hazard ratio 1.52 (95% CI 0.95–2.43)] [5]. Results from a macaque model of SARS-CoV-2 infection also support the use of remdesivir in an early phase of disease [6]

^g^A 10-day course of remdesivir decreased the time to recovery from COVID-19 in the ACTT-1 trial by 31%. This effect was most pronounced in patients that required oxygen therapy [relative risk reduction 1.47 (95% CI 1.17–1.84). The mortality at day 14 was reduced in patients receiving remdesivir [7.1% vs. 11.9%; hazard ratio 0.7 (95% CI 0.47–1.03, *p* value = 0.06)] [1]. In a meta-evaluation of the SIMPLE (GS-US-540-5773) trial and retrospective cohort data, remdesivir treatment was associated with a 62% reduction of the odds ratio for mortality in patients with severe COVID-19 [7]

^h^Comparing the efficacy of a 5-day versus a 10-day course of remdesivir in patients with severe COVID-19 (SIMPLE SEVERE trial) a 10-day course was not superior to a 5-day treatment [8]

^i^Reduced mortality at day 28 by one-third in patients with mechanical ventilation [29.3% vs 41.4%; rate ratio (RR) 0.64 (95% CI 0.72-0.94)] [2]

^j^A clinical benefit of remdesivir for patients with critical COVID-19 is less well documented. In the subgroup of patients with invasive ventilation in the ACTT trial, no effect on the time to recovery was demonstrated [1]. The present recommendation is based on patients with severe COVID-19
